# Effect of ALDH1A1 and CD44 on Survival and Disease Recurrence in Patients With Osteosarcoma

**DOI:** 10.7759/cureus.52404

**Published:** 2024-01-16

**Authors:** Max R Haffner, Augustine M Saiz, Morgan A Darrow, Sean J Judge, Tammy Laun, Aman Arora, Sandra L Taylor, R Lor Randall, Elysia M Alvarez, Steven W Thorpe

**Affiliations:** 1 Orthopedic Surgery, UC (University of California) Davis Health, Sacramento, USA; 2 Pathology and Laboratory Medicine, UC (University of California) Davis Health, Sacramento, USA; 3 Surgery, UC (University of California) Davis Health, Sacramento, USA; 4 Medicine, Oakland University William Beaumont School of Medicine, Auburn Hills, USA; 5 Urology, UC (University of California) Davis School of Medicine, Sacramento, USA; 6 Division of Biostatistics, Public Health Sciences, Sacramento, USA; 7 Pediatric Hematology and Oncology, UC (University of California) Davis Health, Sacramento, USA

**Keywords:** osteosarcoma, aldh1, cd44+, cancer survival, molecular biomarker, osteosarcoma research

## Abstract

Purpose: Emerging evidence suggests that osteosarcoma stem cells (OSCs) may be responsible for tumor initiation propagation, recurrence, and resistance to therapy. We set out to evaluate the relationship between the abundance of ALDH1A1 and CD44-positive cells in biopsy and resection samples on disease recurrence and overall survival.

Methods: A retrospective review of 20 patients, including biopsy and resection samples, was performed at a comprehensive cancer center. Additionally, we queried the publicly available TARGET dataset of osteosarcoma patients.

Results: Neither the percentages of ALDH1A1-positive cells nor CD44-positive cells were significantly associated with overall mortality or disease recurrence in either biopsy or resection samples. Unlike our institutional data, overall survival was significantly correlated to higher ALDH1A1 expression in the TARGET dataset both in univariate and age-adjusted analyses.

Conclusions: ADLH1 and CD44, potential markers of OSCs, were not found to be reliable clinical immunohistochemical prognostic markers for osteosarcoma patient survival, specifically disease-free survival. Osteosarcoma patients with high ALDH1A1 RNA expression showed improved overall survival in examining a national genomic database of osteosarcoma patients but again no association with disease-free survival. The potential of CD44 and ALDH1A1 as cellular-specific prognostic markers of survival, and as possible molecular targets, may be limited in osteosarcoma.

## Introduction

Osteosarcoma (OS) is the most common primary bone tumor and a leading cause of cancer-related death in children and young adults [1,2]. With current treatment protocols, five-year survival rates are approximately 70-80% for localized disease and 20-30% for those presenting with metastatic disease at initial diagnosis [1,3-5]. Despite significant advancements in basic and translational science, survival rates in OS have remained stagnant since the late 1980s [2]. There remains a need for novel therapeutics given the overall poor prognosis with metastatic disease and the lack of significant improvements in the last three decades.

Currently, worse outcomes are associated with high-grade tumors with specific histologic subtypes, large tumors, and poor response to induction chemotherapy, as indicated by a low percentage of tumor necrosis on post-chemotherapy resection pathology [6-8]. However, these factors remain broad prognostic indicators that lack specificity at the individual patient level. More precise cellular and molecular markers of treatment response beyond tumor necrosis are needed.

Cancer stem cells (CSCs) represent a promising prognostic and therapeutic target in OS treatment. CSCs are a potential source for cancer self-renewal and metastasis as they represent the progenitor source for the overall tumor and may represent treatment-resistant clones. Previous literature exploring CSCs in OS has specifically looked at CD44 as a marker for CSCs with mixed results [9,10]. Nonetheless, CD44 silencing via CRISPR-Cas9 in vitro has demonstrated diminished OS invasiveness and enhanced chemosensitivity [11]. Likewise, ALDH1A1, a newer CSC marker, responsible for the oxidation of intracellular aldehydes, has been investigated in several other cancers with variable prognostic findings [12-23]. ALDH1A1 has not been thoroughly evaluated in bone tumors, with limited studies evaluating its role in OS [24,25]. The clinical impact of ALDH1A1 as a prognostic factor or an enzymatic biochemical target for treatment in OS remains to be determined. Aldehyde dehydrogenase (ALDH) has also been implicated in OS lung metastasis microenvironmental interaction. In vitro and clinical trials for repurposing disulfiram for cancer treatment have been considered based on targeting ALDH1A1 [26]. Given these clinical interests, we aimed to evaluate ALDH1A1 and CD44 as prognostic markers in OS in clinical practice using immunohistochemical (IHC) analysis in biopsy and resection specimens.

The purpose of this study was to examine the association between CSC markers ALDH1A1 and CD44 and overall survival, metastasis potential, and disease recurrence in OS patients from both an institutional review and a national database. We hypothesized that the patients with increased CD44 and ALDH1A1 staining on biopsy and/or resection samples would have decreased survival, increased rate of metastasis, and would be more likely to experience disease relapse. We aimed to determine if ALDH1A1 or CD44 could provide clinically relevant tumor-specific prognostic information to aid in counseling patients and direct potential management with ALDH1A1 targeted treatment.

## Materials and methods

Patient characteristics

This was a retrospective review of OS patients treated at the University of California (UC) Davis Comprehensive Cancer Center (UCD) from 2005 to 2015. Twenty patients with biopsy-proven OS were included in the study. A second analysis was conducted using the TARGET-OS public database, which contains a comprehensive clinical and biochemical dataset for a cohort of 85 pediatric OS patients.

Tissue specimens

Two tissue microarrays (TMA) were created from archived specimens, one for biopsies and one for resections. IHC stains for CD44 and ALDH1A1 were performed on each TMA (CD44: MA513890, Thermo Fisher Scientific, Waltham, MA; ALDH1A1: ABIN513238, Abnova, Taipei, Taiwan). The IHC slides were evaluated and scored via two different methods: (1) quantitative image software analysis and (2) semi-quantitative manual review by the study pathologist (MD). The quantitative analysis was performed by Indica Labs using their proprietary HALO software to identify the total percentage of positive cells as well as the stain intensity (weak 1+, moderate 2+, strong 3+) for CD44 and ALDH1A1 in each sample. The study pathologist independently evaluated the same TMA slides to produce the same metrics (total percentage of positive cells and stain intensity). H-scores were calculated as described by Hirsch et al. [27]. The primary outcome measures examined were overall survival and disease-free survival for patients in correlation with CD44 and ALDH1A1 on biopsy and resection samples.

The Cancer Genome Atlas (TCGA) analysis

Clinicodemographic and RNA expression data were retrieved from the publicly available TARGET-OS dataset, using the UCSC Xena platform (University of California Santa Cruz, Santa Cruz, CA) [28]. Normalized RNA expressions of ALDH1A1 and CD44 were matched to each patient. Patients were separated by median RNA expression.

For this analysis, four patients did not have CD44 or ALDH1A1 data. Two patients lacked outcome information (neither survival nor recurrence data). One patient had overall survival data but not recurrence information and was not used in the disease-free survival analysis. Finally, one patient did not have survival time and was omitted. In total, 85 patients from the TARGET dataset were available for analysis.

Statistical analysis

Kaplan-Meier curves were generated for overall survival and disease-free survival separately for the UC Davis and TCGA datasets. The time-to-event for overall survival was the time from diagnosis to death or last follow-up. For disease-free survival, the time-to-event was the time from surgery to disease recurrence, death, or last follow-up. Local or distant recurrence was coded as disease recurrence.

For the UC Davis data, to evaluate the relationship between CD44 and ALDH1A1-positive cell abundance with survival and disease recurrence, the average percentage of all positive cells was calculated for each subject separately for the resection and biopsy samples. An H-score was also calculated for each sample as [1 x (% 1+ cells) + 2 x (% 2+ cells) + 3 x (% 3+ cells)] and averaged for each subject. These averages were used as predictors in proportional hazard models of survival and disease recurrence. Proportional hazard models were also used to evaluate the relationships between manually generated H-scores from resection and biopsy samples with overall survival and disease-free survival.

Similarly, for the TCGA dataset, the effect of CD44 and ALDH1A1 values on overall survival and disease-free survival were evaluated with proportional hazards models. Age was then added as a covariate in both regressions. Statistical analyses were conducted using R statistical computing software version 3.6.3 (R Foundation for Statistical Computing, Vienna, Austria). All statistical tests were two-sided and evaluated at a significance level of 0.05.

## Results

In total, 20 UCD patients were identified for this study (Table 1). For analyses using biopsy data, 18 and 17 patients were available for analysis of overall survival and disease-free survival using the documented time of diagnosis to their last known follow-up. One patient died prior to obtaining a resection sample and two patients lacked biopsy data. For analyses using resection data, 14 patients had available data. One patient died prior to obtaining a resection sample. The other five patients lacked resection data due to the inability to stain samples due to necrosis.

**Table 1:** Characteristics of 20 subjects with osteosarcoma. ALDH = aldehyde dehydrogenase; MAP = methotrexate, doxorubicin, and cisplatin; DOD = died of disease; AWD = alive with disease; NED = no evidence of disease.

Patient #/Sex	Age at diagnosis (years)	Primary location	Local recurrence	Distant metastasis	Neoadjuvant chemotherapy	Resection % necrosis	Length of follow-up (months)	Status	ALDH biopsy H-score	ALDH: % positive cells
1/F	10.6	R tibia	No	Yes	MAP	<3	432.8	AWD	20	12
2/F	13.9	L humerus	n/a	No	MAP	>90	289.3	NED	2	2
3/M	55.6	R femur	No	No	None	0	308.8	NED	12	6
4/F	13.1	L femur	No	n/a	MAP	20	15.3	DOD	9	6
5/M	62.2	L ulna	No	No	MAP	90	305.8	NED	77	47
6/M	13.1	L tibia	No	Yes	MAP	50	383.2	DOD	52	27
7/M	14.3	R femur	Yes	No	MAP	>95	179.8	NED	20	11
8/F	13.9	L femur	No	No	MAP	95	153.9	NED	75	42
9/M	19.4	R fibula	No	Yes	MAP	100	74.4	NED	17	6
10/M	36.9	R femur	No	No	MAP	25	18	NED	17	10
11/M	25.7	R tibia	n/a	No	MAP	60	72	NED	-	-
12/F	11.0	L tibia	No	Yes	MAP	10	24	DOD	34	23
13/F	11.8	L tibia	No	Yes	MAP	Unknown	21.5	DOD	8	7
14/F	20.4	R femur	No	Yes	Unknown	>90	109.3	DOD	75	42
15/M	10.2	R tibia	No	Yes	Herceptin, doxorubicin, dexrazoxane, cisplatin	90	66.8	DOD	112	58
16/M	15.4	Pelvis	No	Yes	Methotrexate, allopurinol	>95	166.5	DOD	18	12
17/M	40.6	L femur	No	Yes	MAP	95	95.7	AWD	13	7
18/F	17.6	L femur	No	Yes	MAP	100	90.8	AWD	12	7
19/F	12.5	L femur	No	No	Unknown	58	190.6	NED	18	12
20/F	11.1	L femur	No	No	Unknown	>99	26.8	NED	-	-

Effect of positive cell counts on overall and disease-free survival

**Figure 1:**
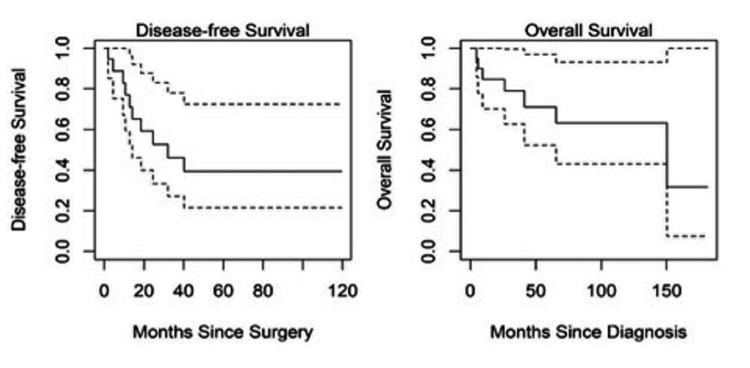
Disease-free and overall survival estimates with 95% confidence intervals. The solid line is the estimated survival and the dotted lines are the upper and lower 95% confidence limits.

**Figure 2:**
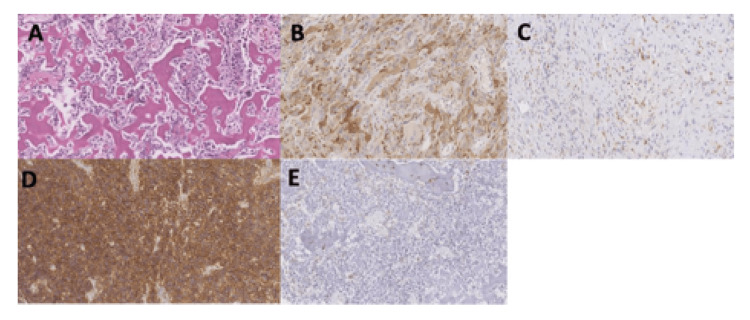
(A) Conventional osteosarcoma histology showing osteoid production by malignant cells. H&E stain image of osteosarcoma with examples of both high and low H-scores of ALDH1 (B, C) and CD44 (D, E). All images are shown at 200x magnification. H&E = hematoxylin and eosin.

Neither the percentages of CD44/ALDH1A1 positive cells nor the H-scores were significantly associated with hazards of overall mortality or disease recurrence. This was true for both biopsy and resection samples based on counts from quantitative image software analysis and semi-quantitative manual review (Tables 2-5). Kaplan-Meier curves for overall survival and disease-free survival are shown in Figure 1. Examples of OS histology and stains for ALDH1A1 and CD44 are shown in Figure 2.

**Table 2:** Estimated hazard ratio (95% confidence limits) and significance testing results for the effect of the percentage of positive cells and H-scores for ALDH1A1 in biopsy and resection samples derived from computer-generated values on overall and disease-free survival based on the proportional hazard model.

	Overall survival		Disease-free survival	
	Hazard ratio	P-value	Hazard ratio	P-value
Biopsy samples				
% positive	1.02 (0.99, 1.05)	0.265	0.99 (0.97, 1.01)	0.521
H-score	1.01 (0.99, 1.03)	0.205	1 (0.98, 1.01])	0.628
Resection samples				
% positive	1.02 (0.99, 1.06)	0.119	1.02 (0.99, 1.05)	0.190
H-score	1.01 (1, 1.03)	0.101	1.01 (1, 1.03)	0.131

**Table 3:** Estimated hazard ratio (95% confidence limits) and significance testing results for the effect of the percentage of positive cells and H-scores for CD44 in biopsy and resection samples derived from computer-generated values on overall and disease-free survival based on the proportional hazard model.

	Overall survival		Disease-free survival	
	Hazard ratio	P-value	Hazard ratio	P-value
Biopsy samples				
% positive	1.01 (0.98, 1.04)	0.543	0.99 (0.97, 1.02)	0.718
H-score	1.01 (0.98, 1.04)	0.535	0.99 (0.97, 1.02)	0.725
Resection samples				
% positive	0.99 (0.95, 1.04)	0.740	0.99 (0.95, 1.03)	0.622
H-score	0.99 (0.95, 1.03)	0.704	0.99 (0.95, 1.03)	0.587

**Table 4:** Estimated hazard ratio (95% confidence limits) and significance testing results for the effect of the percentage of positive cells and H-scores for CD44 in biopsy and resection samples derived from manual counts on overall and disease-free survival based on the proportional hazard model.

	Overall survival		Disease-free survival	
	Hazard ratio	P-value	Hazard ratio	P-value
Biopsy samples				
% positive	1.01 (0.98, 1.05)	0.431	0.99 (0.97, 1.01)	0.422
H-score	1.01 (0.99, 1.02)	0.380	1 (0.99, 1.01)	0.472
Resection samples				
% positive	1.02 (0.99, 1.05)	0.261	1.01 (0.98, 1.04)	0.567
H-score	1.01 (1, 1.02)	0.182	1 (0.99, 1.02)	0.374

**Table 5:** Estimated hazard ratio (95% confidence limits) and significance testing results for the effect of the percentage of positive cells and H-scores for ALDH1A1 in biopsy and resection samples derived from manual counts on overall and disease-free survival based on the proportional hazard model.

	Overall survival		Disease-free survival	
	Hazard ratio	P-value	Hazard ratio	P-value
Biopsy samples				
% positive	1.02 (0.98, 1.06)	0.396	1 (0.97, 1.04)	0.944
H-score	1.01 (0.99, 1.03)	0.432	1 (0.98, 1.02)	0.833
Resection samples				
% positive	0.99 (0.95, 1.04)	0.793	0.99 (0.96, 1.03)	0.740
H-score	1 (0.98, 1.02)	0.875	1 (0.98, 1.02)	0.775

Assessment of the TARGET-OS dataset

**Figure 3:**
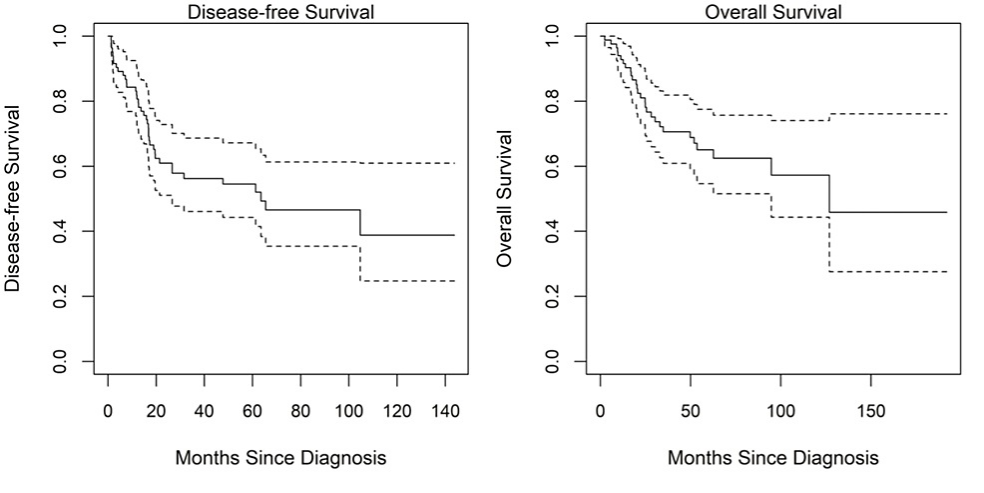
Disease-free and overall survival estimates with 95% confidence intervals for the TARGET dataset. The solid line is the estimated survival and the dotted lines are the upper and lower 95% confidence limits.

Clinicodemographic characteristics and matched RNA expression analysis from resected or biopsied tumors were available for 85 patients. Data summary statistics of subject characteristics are presented in Table 6. Kaplan-Meier curves for overall survival and disease-free survival are shown in Figure 3. High ALDH1A1 expression is associated with improved outcomes using the TARGET-OS dataset.

**Table 6:** Characteristics of 85 subjects with osteosarcoma from the TARGET dataset.

Variable	Summary
Age (mean ± SD)	15.2 ± 4.9
Sex	42% female (n = 36)
Mortality	33% (n = 29)
Recurrence or death	46% (n = 39)

Unlike our institutional data, overall survival was significantly related to ALDH1A1 levels in the TARGET dataset both in univariate and age-adjusted analyses (Table 7). Overall survival increased with increasing ALDH1A1 values. Overall survival and CD44 levels did not have a statistically significant correlation.

**Table 7:** Estimated hazard ratio (95% confidence limits) and significance testing results for the effect of ALDH1A1 and CD44 on survival in the TARGET dataset. ALDH = aldehyde dehydrogenase.

	Overall survival		Disease-free survival	
	Hazard ratio	P-value	Hazard ratio	P-value
Univariate				
ALDH	0.78 (0.64, 0.95)	0.015	0.87 (0.75, 1.02)	0.09
CD44	0.74 (0.54, 1)	0.053	0.85 (0.66, 1.09)	0.194
Age-adjusted				
ALDH	0.78 (0.64, 0.95)	0.016	0.88 (0.75, 1.03)	0.120
CD44	0.74 (0.54, 1)	0.053	0.86 (0.67, 1.09)	0.214

## Discussion

The role of ALDH1A1 and CD44 in OS as biologic markers of a subset of tumor cells that may confer treatment resistance has yet to be clinically evaluated or validated. Our study coupled the IHC identification of ALDH1A1 and CD44 cells in biopsy and resection samples and correlated them with patient survival. Increased ALDH1A1 RNA expression correlated with improved overall survival in our analysis of 85 patients with OS found using the TARGET-OS dataset. Increased CD44 RNA expression trended toward being a negative prognostic indicator in the same cohort. In contrast, we did not find evidence of an association between IHC ALDH1A1 or CD44 protein expression and OS patient prognosis in our institutional cohort of 20 patients. Regardless of the method, neither CD44 nor ALDH1A1 had evidence of any prognostic value regarding disease-free survival.

The value of ALDH1A1 and CD44 as prognostic markers may be limited. Although there was a weak association with improved overall survival using the TARGET-OS dataset, this did not correlate with IHC staining. Clinical implications of our study suggest these markers in OS patients may not be useful for routine clinical use in determining prognosis or targets for treatment. This finding conflicts with basic science and rodent model evidence showing that increased ALDH expression leads to improved cellular resistance to oxidative stress and in turn may lead to increased metastatic potential, drug resistance, and worse prognosis [26]. Further studies evaluating the biochemical pathways CD44 and ALDH1A1 in OS compared to other cancers may further elucidate critical differences that affect tumor behavior.

Outside of OS, there have been several translational and clinical studies demonstrating mixed prognostic outcomes related to increased ALDH expression. Chang et al. studied 442 patients with ovarian cancer and found high ALDH1A1 expression correlated with favorable survival and improved response to chemotherapy [29]. Likewise, Taylor et al. found that ALDH1A1 expression was an independent prognostic indicator for patient survival in 68 patients with malignant melanoma and at least 10 years of follow-up [17]. These studies contrast data implementing ALDH1A1 as a poor prognostic indicator in other cancers [21,30]. As theorized by previous authors, it is likely that the ubiquitous enzyme ALDH1A1 does not serve the same role in CSC activity in all cancer types [17,30].

Within OS, there is limited evidence around ALDH and its prognostic value. Greco et al. published a 2014 retrospective review of 10 bone sarcoma patients (three OS, five chondrosarcoma, and two Ewing sarcoma), reporting a high percentage of ALDH-high cells in all eight patients who had metastatic disease [25]. In the study, two of the three OS patients had just met the threshold that the authors set to be considered a high percentage of ALDH-high cells. More so, these determinations were based on the flow cytometric-based Aldefluor assay, not IHC. Additionally, Honoki et al. found that human OS and fibrosarcoma cells with elevated ALDH1A1 were more resistant to cisplatin and doxorubicin in vitro [24]. To date, the TARGET-OS cohort of 85 patients is the largest to correlate ALDH1A1 and OS prognosis.

We were able to corroborate the TARGET-OS data with our IHC data to strengthen the validity of our results in a small series and evaluate differences between the methodologies. In our study utilizing IHC, there was no association between ALDH1A1 and outcomes. This could be due to our small case series being unable to detect a difference. Additionally, this may be due to variable differences in RNA expression as measured by the TARGET-OS dataset compared to IHC measuring protein presence used at our institution's cohort. Further research into the cellular mechanistic pathways of CSCs, especially regarding CD44 and ALDH1A1, may provide further insight into the optimal cellular prognostic target.

CD44 also has shown conflicting results in its prognostic value related to OS patients. In 2015, Gao et al. examined 114 human OS tumor specimens from primary, metastatic, and recurrent stages, and determined that CD44 was overexpressed in metastatic and recurrent OS as compared with primary tumors with higher expression of CD44 in patients with shorter survival and patients who exhibited unfavorable response to chemotherapy before surgical resection [9]. Contrarily, in 2014, Liu et al. found in their meta-analysis that included six studies with 329 OS patients that CD44 expression was not associated with overall survival rate and metastasis in OS [10].

A limitation of this study includes the small sample size at a single institution. OS is a rare cancer with only a prevalence of 1% of all cancer diagnoses [31]. Our institutional cohort was small with only sufficient power to detect large effects. Additionally, our staining methods, although standardized to our pathology laboratory, may not be applicable to other centers around the world or directly comparable to the TARGET-OS dataset. Finally, although our treatment guidelines are in line with the National Comprehensive Center Network, each individual case presents different challenges and opportunities that are also affected by the treating physician’s discretion, so identical treatment and therefore responses cannot be obtained. The strength of our study is the novelty of investigating ALDH1A1 in OS in an IHC TMA-based approach and correlating this to biopsy-proven OS patients’ outcomes and correlating with the TCGA data.

## Conclusions

ADLH1 and CD44 are not reliable clinical prognostic markers for OS patient survival, specifically disease-free survival. OS patients with high ALDH1A1 RNA expression showed improved overall survival in examining a national genomic database of OS patients but again without association with disease-free survival. The potential of CD44 and ALDH1A1 as cellular-specific prognostic markers of survival, and as possible molecular targets, may be limited. The continued search for new cellular treatment response surrogates remains critical to advance beyond nonspecific tumor necrosis as the primary prognostic marker. Ideally, future cellular mechanistic markers will give physicians a quantifiable measure to assess patient response to treatment, predict recurrence risk and patient survival, and guide patient counseling and tumor-specific treatment. Further mechanistic studies are needed to better understand CD44 and ALDH1A1 activity in OS.

## References

[1] Isakoff MS, Bielack SS, Meltzer P, Gorlick R (2015). Osteosarcoma: current treatment and a collaborative pathway to success. J Clin Oncol.

[2] Mirabello L, Troisi RJ, Savage SA (2009). Osteosarcoma incidence and survival rates from 1973 to 2004: data from the Surveillance, Epidemiology, and End Results Program. Cancer.

[3] Meyers PA, Schwartz CL, Krailo M (2005). Osteosarcoma: a randomized, prospective trial of the addition of ifosfamide and/or muramyl tripeptide to cisplatin, doxorubicin, and high-dose methotrexate. J Clin Oncol.

[4] Ferrari S, Meazza C, Palmerini E (2014). Nonmetastatic osteosarcoma of the extremity. Neoadjuvant chemotherapy with methotrexate, cisplatin, doxorubicin and ifosfamide. An Italian Sarcoma Group study (ISG/OS-Oss). Tumori.

[5] Duchman KR, Gao Y, Miller BJ (2015). Prognostic factors for survival in patients with high-grade osteosarcoma using the Surveillance, Epidemiology, and End Results (SEER) Program database. Cancer Epidemiol.

[6] Hauben EI, Weeden S, Pringle J, Van Marck EA, Hogendoorn PC (2002). Does the histological subtype of high-grade central osteosarcoma influence the response to treatment with chemotherapy and does it affect overall survival? A study on 570 patients of two consecutive trials of the European Osteosarcoma Intergroup. Eur J Cancer.

[7] Friebele JC, Peck J, Pan X, Abdel-Rasoul M, Mayerson JL (2015). Osteosarcoma: a meta-analysis and review of the literature. Am J Orthop (Belle Mead NJ).

[8] Marina NM, Smeland S, Bielack SS (2016). Comparison of MAPIE versus MAP in patients with a poor response to preoperative chemotherapy for newly diagnosed high-grade osteosarcoma (EURAMOS-1): an open-label, international, randomised controlled trial. Lancet Oncol.

[9] Gao Y, Feng Y, Shen JK (2015). CD44 is a direct target of miR-199a-3p and contributes to aggressive progression in osteosarcoma. Sci Rep.

[10] Liu Y, Wu Y, Gu S (2014). Prognostic role of CD44 expression in osteosarcoma: evidence from six studies. Diagn Pathol.

[11] Xiao Z, Wan J, Nur AA, Dou P, Mankin H, Liu T, Ouyang Z (2018). Targeting CD44 by CRISPR-Cas9 in multi-drug resistant osteosarcoma cells. Cell Physiol Biochem.

[12] Zhong Y, Shen S, Zhou Y (2014). ALDH1 is a better clinical indicator for relapse of invasive ductal breast cancer than the CD44+/CD24- phenotype. Med Oncol.

[13] Zhou C, Sun B (2014). The prognostic role of the cancer stem cell marker aldehyde dehydrogenase 1 in head and neck squamous cell carcinomas: a meta-analysis. Oral Oncol.

[14] Zhou F, Mu YD, Liang J (2015). Aldehyde dehydrogenase 1: a specific cancer stem cell marker for human colorectal carcinoma. Mol Med Rep.

[15] Zhou L, Yu L, Zhu B, Wu S, Song W, Gong X, Wang D (2016). Metastasis-associated in colon cancer-1 and aldehyde dehydrogenase 1 are metastatic and prognostic biomarker for non-small cell lung cancer. BMC Cancer.

[16] Xie Q, Liang J, Rao Q (2016). Aldehyde dehydrogenase 1 expression predicts chemoresistance and poor clinical outcomes in patients with locally advanced cervical cancer treated with neoadjuvant chemotherapy prior to radical hysterectomy. Ann Surg Oncol.

[17] Taylor LA, Abraham RM, Tahirovic E (2017). High ALDH1 expression correlates with better prognosis in tumorigenic malignant melanoma. Mod Pathol.

[18] Tamatani T, Takamaru N, Ohe G, Akita K, Nakagawa T, Miyamoto Y (2018). Expression of CD44, CD44v9, ABCG2, CD24, Bmi-1 and ALDH1 in stage I and II oral squamous cell carcinoma and their association with clinicopathological factors. Oncol Lett.

[19] Nakahata K, Uehara S, Nishikawa S, Kawatsu M, Zenitani M, Oue T, Okuyama H (2015). Aldehyde dehydrogenase 1 (ALDH1) is a potential marker for cancer stem cells in embryonal rhabdomyosarcoma. PLoS One.

[20] Mohamed SY, Kaf RM, Ahmed MM, Elwan A, Ashour HR, Ibrahim A (2019). The prognostic value of cancer stem cell markers (Notch1, ALDH1, and CD44) in primary colorectal carcinoma. J Gastrointest Cancer.

[21] Jiang F, Qiu Q, Khanna A (2009). Aldehyde dehydrogenase 1 is a tumor stem cell-associated marker in lung cancer. Mol Cancer Res.

[22] Huang HH, Wang YC, Chou YC, Yu MH, Chao TK (2018). The combination of aldehyde dehydrogenase 1 (ALDH1) and CD44 is associated with poor outcomes in endometrial cancer. PLoS One.

[23] Chen MF, Chen PT, Lu MS, Chen WC (2018). Role of ALDH1 in the prognosis of esophageal cancer and its relationship with tumor microenvironment. Mol Carcinog.

[24] Honoki K, Fujii H, Kubo A, Kido A, Mori T, Tanaka Y, Tsujiuchi T (2010). Possible involvement of stem-like populations with elevated ALDH1 in sarcomas for chemotherapeutic drug resistance. Oncol Rep.

[25] Greco N, Schott T, Mu X (2014). ALDH activity correlates with metastatic potential in primary sarcomas of bone. J Cancer Ther.

[26] Belayneh R, Weiss K (2020). The role of ALDH in the metastatic potential of osteosarcoma cells and potential ALDH targets. Adv Exp Med Biol.

[27] Hirsch FR, Varella-Garcia M, Bunn PA Jr (2003). Epidermal growth factor receptor in non-small-cell lung carcinomas: correlation between gene copy number and protein expression and impact on prognosis. J Clin Oncol.

[28] (2020). UCSC Xena. https://xena.ucsc.edu/.

[29] Chang B, Liu G, Xue F, Rosen DG, Xiao L, Wang X, Liu J (2009). ALDH1 expression correlates with favorable prognosis in ovarian cancers. Mod Pathol.

[30] Bi X, Wu C, Han M, Cai J (2012). Correlations of ALDH1 expression with molecular subtypes and ABCG2 in breast cancer. Gland Surg.

[31] Anderson ME (2016). Update on survival in osteosarcoma. Orthop Clin North Am.

